# High-CO_2_ Modified Atmosphere Packaging with Superchilling (−1.3 ^°^C) Inhibit Biochemical and Flavor Changes in Turbot (*Scophthalmus maximus*) during Storage

**DOI:** 10.3390/molecules25122826

**Published:** 2020-06-19

**Authors:** Jun Mei, Feng Liu, Shiyuan Fang, Weiqing Lan, Jing Xie

**Affiliations:** 1 College of Food Science and Technology, Shanghai Ocean University, Shanghai 201306, China; jmei@shou.edu.cn (J.M.); m170200413@st.shou.edu.cn (F.L.); m170200401@st.shou.edu.cn (S.F.); wqlan@shou.edu.cn (W.L.); 2 National Experimental Teaching Demonstration Center for Food Science and Engineering Shanghai Ocean University, Shanghai 201306, China; 3 Shanghai Engineering Research Center of Aquatic Product Processing and Preservation, Shanghai 201306, China; 4 Shanghai Professional Technology Service Platform on Cold Chain Equipment Performance and Energy Saving Evaluation, Shanghai 201306, China

**Keywords:** turbot, modified atmosphere packaging, superchilling, shelf life, quality

## Abstract

The effects of modified atmosphere packaging (MAP) in combination with superchilling (−1.3 °C) on the physicochemical properties, flavor retention, and organoleptic evaluation of turbot samples were investigated during 27 days storage. Results showed that high-CO_2_ packaging (70% or 60% CO_2_) combined with superchilling could reduce the productions of off-flavor compounds, including total volatile basic nitrogen (TVB-N) and ATP-related compounds. Twenty-four volatile organic compounds were determined by gas chromatography–mass spectrometry (GC/MS) during storage, including eight alcohols, 11 aldehydes, and five ketones. The relative content of off-odor volatiles, such as 1-octen-3-ol, 1-penten-3-ol, (E)-2-octenal, octanal, and 2,3-octanedione, was also reduced by high-CO_2_ packaging during superchilling storage. Further, 60% CO_2_/10% O_2_/30% N_2_ with superchilling (−1.3 °C) could retard the water migration on the basis of the water holding capacity, low field NMR, and MRI results, and maintain the quality of turbot according to organoleptic evaluation results during storage

## 1. Introduction

Turbot (*Scophthalmus maximus*) is a flat fish species with high economic value and nutritional benefits and widely cultivated in China [1]. Fish freshness is crucial for fish quality and directly determines the consumer acceptability and ultimate commercial value of fish [2]. Traditionally, fish freshness evaluation has been based on organoleptic, chemical, and microbiological methods. The quality deterioration of fish after death results from microbiological spoilage and chemical reactions, such as changes in protein and lipid fractions, and the formation of biogenic amines. Some spoilage indicators have been used to evaluate the quality of fish including total volatile base nitrogen (TVB-N), trimethylamine (TMA) and biogenic amine composition, which can be performed by microbiological count and identification [3]. Turbot is extremely susceptible to endogenous enzymes and exogenous spoilage bacteria, for example, H_2_S-producing bacteria, *Pseudomonas* spp., and *Aeromonas* spp [4,5]. Considering that psychotropic microorganisms can survive and proliferate at cold temperatures, the microbiological safety of fresh turbot should be controlled through inhibiting or delaying of microbiological growth.

Superchilling is the process of lowering the temperature of a product just below its initial freezing temperature, and thereby approximately 5–30% of water is preserved frozen within the food products [6,7]. Superchilling has been used in fish processing to increase the shelf life and has been successfully applied in the preservation of Atlantic mackerel [8], hairtail [9], olive flounder [10], seabream [11], as well as other seafood products. Besides the temperature control, MAP could also inhibit the growth of spoilage bacteria on fish. Zhu et al. [12] reported that superchilling (−0.7 °C) with high-CO_2_ packaging (60% CO_2_/40% N_2_) could substantially inhibit the biochemical and microbiological deterioration of catfish (*Clarias gariepinus*) muscle during storage. Parlapani et al. [13] reported that 2 °C combined with 60% CO_2_/10% O_2_/30% N_2_ could slow down the TVB-N and TMA-N increase and extend the shelf life by about four days.

The objective of this research was to assess the potential benefits of superchilling at −1.3 °C combined with different high-CO_2_ MAP systems by monitoring the biochemical changes, determining the volatile compounds using GC/MS, and evaluating the organoleptic properties during storage.

## 2. Results and Discussion

The turbot samples were considered spoiled and no further sampling was performed when the organoleptic evaluation results declined below 3.0. Therefore, the last sampling point for air packaging (AP), vacuum packaging (VP), and MAP treated samples was at day 21, day 24, and day 27, respectively.

### 2.1. Water Holding Capacity (WHC)

**Figure 1:**
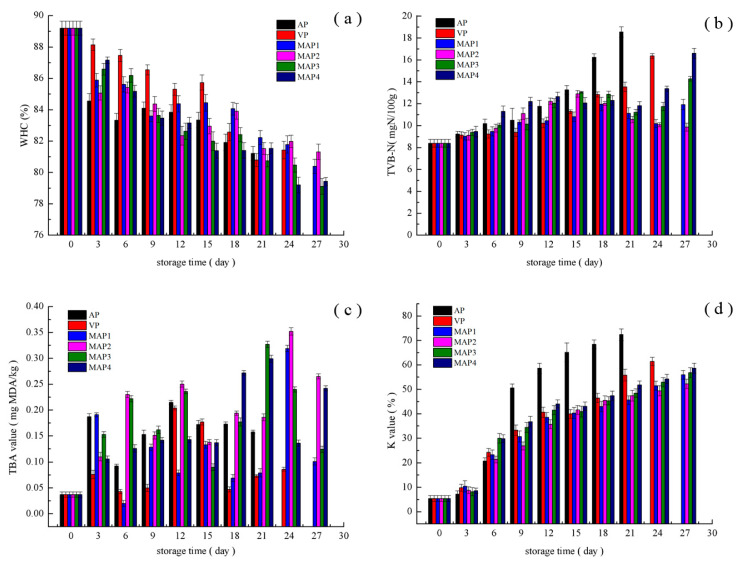
Changes in water holding capacity (WHC) (**a**), TVB-N value (**b**), TBA value (**c**) and K value (**d**) of samples under different treatments during storage period (AP: air packing; VP: vacuum packing; MAP1: 70% CO_2_/30% N_2_; MAP2: 60% CO_2_/10% O_2_/30% N_2_; MAP3: 50% CO_2_/15% O_2_/35% N_2_; MAP4: 55% CO_2_/5% O_2_/40% N_2_).

As shown in Figure 1a, the WHC of all turbot samples showed a declining trend during storage. This could be attributed to the advancement of protein denaturation that reflects a decrease in the water-protein interactions with increasing storage time [14,15]. The initial WHC value of turbot samples was 89.20%, and AP, VP, MAP1, MAP2, MAP3, and MAP4 treated samples reached significantly (*p* < 0.05) to 81.91%, 82.57%, 84.06%, 83.90%, 82.41%, and 81.4%, at day 18, respectively. From day 0 to 15, VP treated samples had higher WHC values than other turbot samples, which indicated that turbot samples under the VP treatments combined with superchilling have an advantage in maintaining water content from the beginning to middle stages of storage. Decreased WHC in VP treated samples may be related to the myosin denaturation during storage. However, MAP1 and MAP2 treated samples had significantly higher (*p* < 0.05) WHC values than others after day 18 as the higher CO_2_ MAP treatments were more effective in preventing the protein degradation during superchilling storage.

### 2.2. Total Volatile Base Nitrogen (TVB-N) Production

TVB-N mainly concerns the degradation of protein and non-protein nitrogenous components generated by spoilage bacteria and enzymes and is extensively used as an indicator for the spoilage degree of marine fish [16,17]. Changes in TVB-N values of turbot samples during storage are summarized in Figure 1b. The TVB-N value of a fresh turbot sample was 8.39 mg/100 g fish muscle, indicating a high standard of turbot samples freshness [18]. The TVB-N values in AP treated samples increased much faster than others due to the increased bacterial activity. The MAP treated samples could reduce the rate of formation of volatile bases as higher concentration of CO_2_ or lower O_2_ inhibited the growth of spoilage bacteria [16,19]. In addition, TVB-N values in VP treated samples were lower than that of MAP treated samples from day 0 to day 15. From day 18, MAP treated samples had lower TVB-N values than VP treated samples and MAP2 presented the lowest TVB-N values. Generally, the upper acceptable level of TVB-N for marine fish is 30 mg/100 g [17], however, all TVB-N values remained below 30 mg/100 g in the present study at the end of storage, which reasserts the efficacy of high CO_2_ and low O_2_ in MAP treatments with superchilling could reduce TVB-N production during storage [12].

### 2.3. Evaluation of Thiobarbituric Acid Reactive Substances (TBARS) Values

The TBARS test measures secondary products of lipid oxidation, primarily malonaldehyde (MDA), including unsaturated carbonyls [20]. The initial TBARS value in the turbot sample was 0.037 mg MDA/kg. During the first 15 days of storage, the TBARS values in all turbot samples showed increasing trends and afterwards slightly decreased due to the reaction of MDA with aldehydes and ketones [21]. The VP treated samples had the lowest TBARS values, and the values of TBARS were significantly affected (*p* < 0.05) by the level of O_2_ in MAP treated samples. TBARS occurred rather different rates with storage time increasing and TBARS values of the AP treated samples were significantly higher (*p* < 0.05) than those of the VP and MAP treated samples at the beginning, indicating that lower O_2_ concentration combined with superchilling could impede the lipid oxidation [22].

### 2.4. K Values

Inosine 5′-monophosphate (IMP) provides a sweet and meaty flavor to improve the quality of fish, however, its transformation to inosine (HxR) and hypoxanthine (Hx) results in unpleasant bitterness reflecting the initial stage of autolytic degradation and subsequent microbiological spoilage. K value is strongly affected by the transformation rate of HxR and Hx and commonly used to evaluate the freshness of fish [23]. The K value of fresh turbot samples was 5.38% and increased during storage. The K value of AP treated samples reached 65.31%, exceeding the rejection limit of 60% at day 15. However, the VP and MAP treatments significantly delayed the increase of K-value (*p* < 0.05), which remained below the rejection limit until the end of storage (day 27). These results indicated that VP or high CO_2_ concentration combined with superchilling could suppress the degradation of ATP and keep the quality of turbot samples at acceptable levels during storage.

### 2.5. Water Distribution by Low Field Nuclear Magnetic Resonance (LF-NMR) Analysis

**Figure 2:**
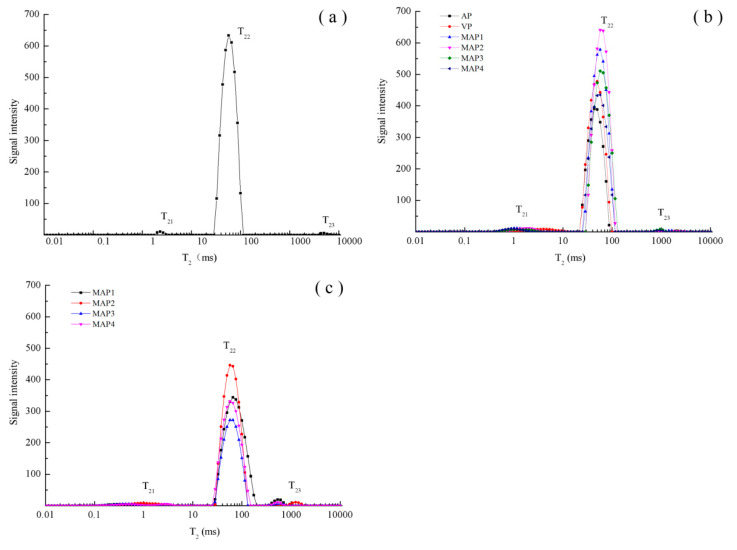
T_2_ relaxation time distribution curves of turbots under different treatments on the 0 (**a**), 15th (**b**) and 27th (**c**) day of the storage period (T_21_, bound water; T_22_, immobilized water; T_23_, free water; AP: air packing; VP: vacuum packing; MAP1:70% CO_2_/30% N_2_; MAP2: 60% CO_2_/10% O_2_/30% N_2_; MAP3: 50% CO_2_/15% O_2_/35% N_2_; MAP4: 55% CO_2_/5% O_2_/40% N_2_).

Low field nuclear magnetic resonance (LF-NMR) is an efficient way to evaluate the freshness of marine fish. T_2_ relaxation method was used to research the proton relaxation behavior and T_21_, T_22_ and T_23_ represent for the bound water, immobile water and free water, respectively. The pT_21_, pT_22_ and pT_23_ were corresponded to the three types of water. pT_21_ did not change significantly (*p* > 0.05) during storage (Figure 2), which was due to this water entrapped within highly organized myofibril structures [24]. pT_22_ diminished progressively during storage (*p* < 0.05) and pT_23_ increased constantly. In the current study, the AP treated samples had lower immobilized water (from 98.62% at day 0 to 95.04% at day 15) than those of other samples. There was no significant difference in the content of immobilized water in MAP1 and MAP2 treated samples (*p* > 0.05). Some studies also demonstrated that water entrapped within myofibrillar released or translated to free water based on the destruction of muscle fiber [25,26]. In addition, this process of water migration also confirmed that MAP treatments retarded the change rates of T_22_.

### 2.6. Analysis of Magnetic Resonance Imaging (MRI)

**Figure 3:**
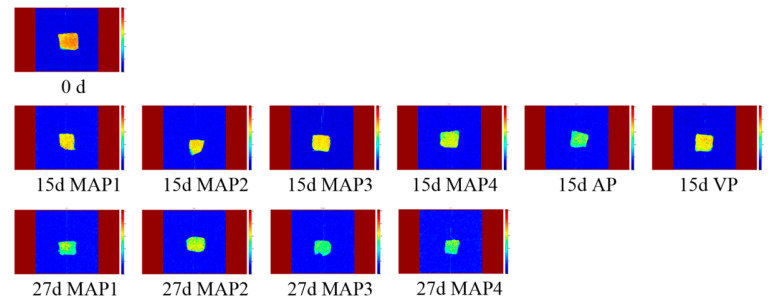
Results of magnetic resonance imaging (MRI) of samples under different treatments during storage period (AP: air packing; VP: vacuum packing; MAP1:70% CO_2_/30% N_2_; MAP2: 60% CO_2_/10% O_2_/30% N_2_; MAP3: 50% CO_2_/15% O_2_/35% N_2_; MAP4: 55% CO_2_/5% O_2_/40% N_2_).

MRI provides visual information for turbot samples during storage. A red color represents a high proton density and blue color stands for low proton density in the pseudo-color images. As shown in Figure 3, the brightness of images varied obscure in the early storage and the brightness of samples became darker and bluer during superchilling storage. At day 15, the color of AP treated samples was bluer and darker than that of other samples, which indicates that the degradation of myofibill and destruction of microstructure in AP treated samples [15], while no visible difference was observed among VP and MAP treated samples. The brightness of MAP2 treated samples is lighter compared to other samples at day 27, which indicated that the MAP2 treatment (60% CO_2_/10% O_2_/30% N_2_) is more suitable for the quality maintenance of turbot and the result was consistent with the variation of LF-NMR transverse relaxation.

### 2.7. Analysis of Free Amino Acids (FAAs)

FAAs are precursors of volatile flavor compounds and biogenic amines [27]. In the present study, the contents of Asp, Thr, Glu, Ala, Met, Ile, Leu, Tyr, Phe, His, Arg, Pro, and total FAAs increased at the beginning and afterwards gradually decreased (Table 1). The MAP treatments retarded the changes of some special FAAs, including Val, Met, Ile, Leu, Phe, Lys, and His. At the same time, Ala and Gly were the most abundant FAAs in turbot samples at day 15 and the AP treated samples had a higher concentration than that of VP and MAP treated samples. His increased from initial value of 1.91 mg/100 g to 9.65, 8.23, 7.45, 4.98, 6.14, and 6.24 mg/100 g at day 15 for AP, VP, MAP1, MAP2, MAP3, and MAP4, respectively. Then His decreased at the end of storage as the precursor of VOCs [28]. Good acceptance of MAP2 treated samples can be explained by its higher Glu and Asp concentrations than others except day 15 during storage and the two FAAs are responsible for stronger umami taste [29]. The MAP treatments could select different predominate bacteria and affect their growth rate and metabolic activity, which can illuminate the difference on FAAs with different treated turbot samples during storage. Overall, the above results demonstrated that flavor deterioration was associated with the decrease of special flavor-enhancing amino acids and accumulation of flavor-detracting amino acids, and MAP combined with superchilling could effectively slow down the process and maintain a good edible value of turbot samples during storage.

### 2.8. Volatile Organic Chemicals (VOCs) Profile

The changes of the main 24 VOCs in turbot samples during storage are presented in Table 1. The major VOCs were C6-C10 alcohols and aldehydes, such as 2-octen-1-ol, 1-octen-3-ol, 1-hexanol, 1-heptanol, 2-ethyl-2-hexen-1-ol, (*E*)-2-octen-1-ol, hexanal, (*Z*)-4-heptenal, heptanal, (*E*,*E*)-2,4-heptadienal, octanal, (*E*)-2-octenal, nonanal, (*E*,*Z*)-2,6-nonadienal, and decanal (Table 2).

Aldehyde has low threshold values and the abundance of aldehyde increased in all turbot samples during storage. Some aldehyde generated from lipid oxidation, especially for the unsaturated fatty acids. Therefore, VOCs generated from the process of lipid oxidation are generally recognized as one of the leading causes of quality deterioration in aquatic products [40]. Hexanal, (*Z*)-4-heptenal, heptanal, (*E*,*E*)-2,4-heptadienal, octanal and (*E*)-2-octenal showed tendencies to increase gradually, while 2-methyl-butanal, nonanal and decanal showed downward trends of turbot samples during storage. Hexanal was generated from the oxidation of linoleic acid and presented in much higher concentrations in the all turbot samples during storage [41]. Further, heptanal, octanal and nonanal might impart a characteristic fishy flavor; however, 2-methyl-butanal renders turbot samples nutty/malty nuances [42]. Benzaldehyde plays a positive effect on fish odor, and a higher relative abundance appeared at the beginning, which was in agreement with Tan et al. [43].

Unsaturated alcohols, such as 1-octen-3-ol, 1-penten-3-ol, 2-octyn-1-ol, (*Z*)-2-penten-1-ol and (*E*)-2-octen-1-ol, have much lower threshold than the saturated alcohols [42]. The most abundant alcohol were 1-penten-3-ol and 1-octen-3-ol with odor like fishy and grassy [44], which are the products of lipoxygenases on ω-3 PUFA [45], and they both significantly increased during storage, especially for the AP treated samples. 1-Octen-3-ol was basically perceived as off-flavor due to the low odor threshold and usually classified as an oxidative spoilage marker [46]. 2-Octen-1-ol imparts a green flavor note and was the product of 12-lipoxygenase activity on ω-6PUFA in turbot samples during storage [47]. The nonbranched alcohols (1-heptanol and 1-hexanol), resulting in a grassy or woody flavor, decreased during storage and 1-heptanol was not detected in MAP treated samples at the end of storage.

Ketones are mainly generated from lipid-autoxidation and/or amino acid degradation, and are related with the unpleasant odor in fish [48]. The main ketone is 2, 3-octanedione, which is produced from linolenate oxidative degradation increased during storage, especially for the AP treated samples, and contributed to the development of oxidized fat-odors [49].

### 2.9. Microbiological Analysis

**Figure 4:**
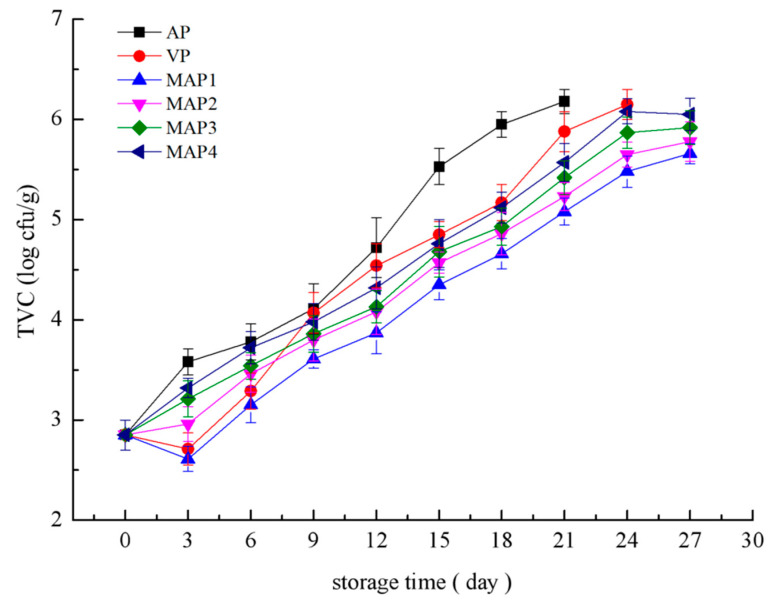
Changes in total viable counts (TVC) of samples under different treatments during storage period (AP: air packing; VP: vacuum packing; MAP1:70% CO_2_/30% N_2_; MAP2: 60% CO_2_/10% O_2_/30% N_2_; MAP3: 50% CO_2_/15% O_2_/35% N_2_; MAP4: 55% CO_2_/5% O_2_/40% N_2_).

It is known that the quality deterioration of fish is mainly caused by microbiological activity, and the total viable count (TVC) variations are well correlated with the changes of sensorial quality score, TVB-N, and K value. Figure 4 shows the data corresponding to the TVC growth of turbot samples at −1.3 °C during storage. The initial population of mesophiles was 2.85 logCFU/g, indicating a good quality of the starting turbot samples with the proposed upper limit of 5 × 10^5^ logCFU/g for fresh fish [30]. The TVC counts increased during storage and the mesophile number of AP treated samples quickly increased to 6.18 log CFU/g at day 21, which exceeded the upper acceptability limit (6 logCFU/g) for marine fish, whereas the MAP1 samples were significantly lower than others during the entire storage. A higher CO_2_ and lower O_2_ concentration could inhibit the microbiological growth [31], which corroborates our microbiological results. Moreover, all the MAP treated samples did not exceed the allowed maximum limit because of the remarkable inhibitory effect of MAP treatments combined with superchilling (−1.3 °C). Some studies have also showed similar results and demonstrated that MAP treatments and superchilling could slow down the microbiological growth [32,33].

### 2.10. Organoleptic Evaluation

**Figure 5:**
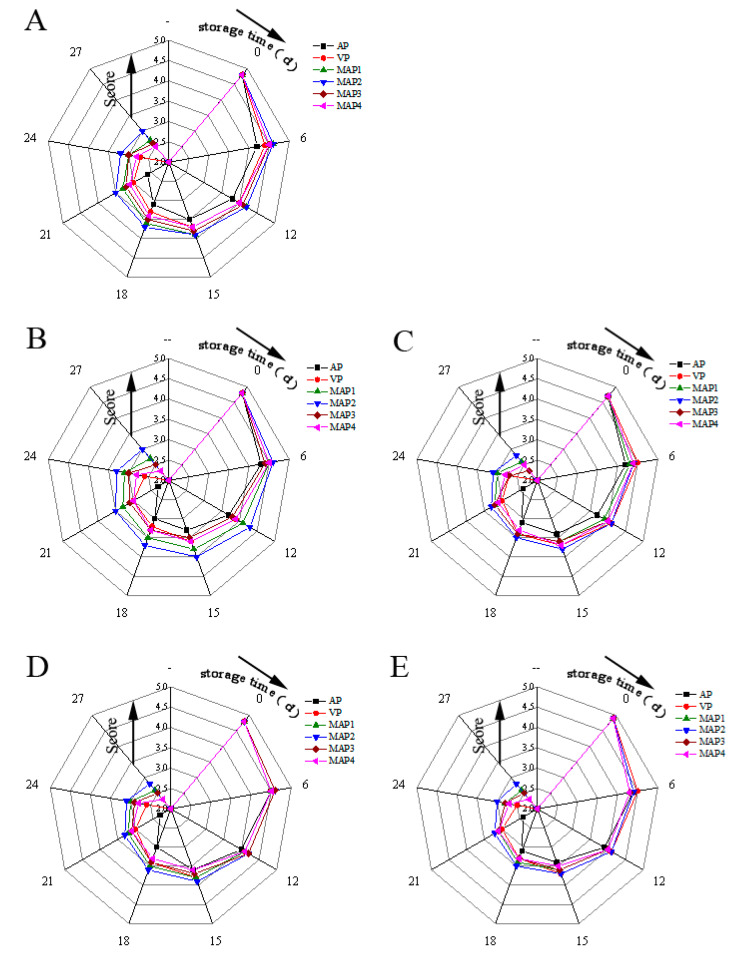
Organoleptic evaluation results of odor (**A**), color (**B**), mucus (**C**), elasticity (**D**) and tissue morphology (**E**) of turbot under different treatments during storage period(AP: air packing; VP: vacuum packing; MAP1: 70% CO_2_/30% N_2_; MAP2: 60% CO_2_/10% O_2_/30% N_2_; MAP3: 50% CO_2_/15% O_2_/35% N_2_; MAP4: 55% CO_2_/5% O_2_/40% N_2_).

The acceptability of turbot samples during superchilling storage depends upon the changes in their organoleptic characteristics. Figure 5 displays organoleptic evaluation results for the turbot samples including odor, color, mucus, elasticity and tissue morphology during superchilling storage. At the beginning, all turbot samples had high organoleptic scores and they were of excellent quality, and then a significant quality loss in all turbot samples was observed (*p* < 0.05) during storage. However, MAP treated samples had significantly higher organoleptic scores than those of the AP and VP treated samples (*p* < 0.05). The AP, VP, MAP3, and MAP4 treated samples were considered unacceptable by the panelist at day 18, day 21, day 25 and day 23, respectively, due to off-odor and loose elasticity. No significant difference (*p* > 0.05) was detected between MAP3 and MAP4. The best organoleptic evaluations were reported in MAP1 and MAP2, however the mucus and odor qualities of MAP1 were unacceptable at day 27. The organoleptic evaluation results were also supported by TVC growth, TVB-N and K values, which were also certified in some researches [34].

## 3. Materials and Methods

### 3.1. Preparation and Treatment of Turbot Samples

A total of 134 live turbot (*Scophthalmus maximus*) fish with an average weight of 500 ± 50 g and length of 30 ± 2 cm were supplied by a local aquatic product market in Luchao Port town (Shanghai, China). They were slaughtered by immersion in ice cold water for 15 min and the gills and viscera of turbot were removed. Then, they were thoroughly washed with sterilized 1% NaCl solutions and 2 random turbot samples were taken to determine the basic quality profiles at initial sampling point (day 0). The remaining turbot samples were divided into six batches (22 fish per packaging condition) for AP, VP, and MAP (70% CO_2_/30% N_2_: MAP1; 60% CO_2_/10% O_2_/30% N_2_: MAP2; 50% CO_2_/15% O_2_/35% N_2_: MAP3; 55% CO_2_/5% O_2_/40% N_2_: MAP4), respectively. For MAP, turbot samples (1 turbot per package) were packaged in polystyrene trays (30 cm × 40 cm × 5 cm) with a gas mixture using a MAP machine (MAP-JY600A, Jiyi Machinery Co., Ltd., Shanghai, China). For AP, 1 turbot in each tray was overwrapped with polyvinylchloride film. Finally, for VP, 1 turbot in each tray was packaged in polyethylene bag and performed at a pressure of –1 bar using a VP machine (DZ-260, Jiahe Packaging Machinery Co., Ltd., Shanghai, China). Then the packaged turbot samples were placed on plastic trays and introduced a freezing tunnel previously cooled to −30 ± 2 °C by air convection. They were kept for 20 min to reach −1.0 °C in the center of the turbot samples. After that, the turbot samples were transferred to a refrigerator (BPS-250CB, Yiheng Thermostatic Chamber, Shanghai, China) and kept at −1.3 ± 0.1 °C. A quality evaluation of turbot samples was performed at 3-day intervals during storage until they were considered spoiled based on the offensive odors by organoleptic evaluation. Three fish were taken out at the sampling point for biochemical changes, VOCs, and organoleptic properties during storage.

### 3.2. Determination of WHC

WHC was determined according to Zang et al. [50]. Then, 3g (m_1_) turbot samples were wrapped in filter paper and then centrifuged at 3000 × *g* for 10 min. After the surface water was drained, the sample was weighed again (m_2_). The WHC was calculated as follows:

$$\mathrm{WHC}\left(\%\right)=\left(1-\frac{m_{1}-m_{2}}{m_{1}}\right)\times 100\%$$

where *m*_1_ is the initial weight (g) of the sample and *m*_2_ is the sample weight (g) after centrifugation.

### 3.3. Determination of TVB-N

For TVB-N determinations, the distillation method of a deproteinized sample as recommended by Djamal et al. [35] was used. Then, 15 g turbot muscle was homogenized in 30 mL of 7.5% TCA solution, centrifuged at 11,620 × *g* for 20 min at 4 °C and the supernatants filtered with Whatman No. 3 qualitative filter paper. Steam distillation was performed with a Kjeldahl nitrogen-determination apparatus (Kjeltec8400, Foss, Denmark) and TVB-N was expressed as mg N/100g.

### 3.4. Determination of TBARS

Lipid oxidation in turbot samples was determined using the evaluation of TBARS test according to Cheng et al. [36] with some modifications. Thereby, 5 g turbot muscle was homogenized in 20 mL of 20 % TBA solution and 20 mL of distilled water, centrifuged at 11,620× *g* for 10 min at 4 °C and the supernatants filtered with Whatman No. 3 qualitative filter paper. The filtrate was diluted with distilled water to 50 mL. 10 mL diluent and 10 mL TBA solution was mixed and heated at 95 °C for 15 min and then cooled with running water. The absorbance of the supernatant was measured at 532 nm using a spectrophotometer (Evolution 220, Thermo, Thermo Fisher Scientific, MA, USA). A standard curve was prepared using 1,1,3,3-tetramethoxypropane at a concentration ranging from 0 to 10 ppm, and the amounts of TBA-RS were expressed as mg of MDA/kg of sample.

### 3.5. Determination of K Value

Adenosine triphosphate (ATP) and its degradation products (adenosine diphosphate (ADP), adenosine monophosphate (AMP), IMP, HxR and Hx) in turbot samples were determined by a RP-HPLC procedure proposed by Li et al. [37]. After obtaining all of the ATP metabolites from HPLC, the freshness index K value was calculated as follows:

(1) $$K\;\mathrm{value}\;\left(\%\right)=\frac{\mathrm{HxR}+\mathrm{Hx}}{\mathrm{ATP}+\mathrm{ADP}+\mathrm{AMP}+\mathrm{IMP}+\mathrm{HxR}+\mathrm{Hx}}\times 100$$

### 3.6. LF-NMR Analysis

The proton relaxation experiments were performed proposed by Li et al. [25] to evaluate the water distribution and migration in turbot samples using a LF-NMR analyzer (MesoMR23-060H.I, Niumag Instrument, Suzhou, China) with a proton resonance frequency of 20 MHz, corresponding to the pulse sequence of Carr-Purcell-Meiboom-Gill (CPMG). The samples from the dorsal part of turbot samples were cut into small squares (1 × 1 × 1 cm) and sealed with polyethylene films. 16 scans were performed with 3000 echoes for each measurement; the relative content of three types of water components was obtained from the iterative inversion with analytical software of T_2_ transverse relaxation time. Acquisition parameters were as follows: slice width = 3 mm, time repetition (TR) = 2000, and time echo (TE) = 15 ms.

MRI experiments were performed to get proton density weighted images and the echo time, repetition time and slice width were 18.2 ms, 850 ms, and 2 mm, respectively.

### 3.7. Headspace SPME-GC/MS Analysis

The VOCs of turbot samples were determined using the method portrayed by Li et al. [38]. Thereby, 3 g minced muscle samples and 6 mL saturated NaCl solution were transferred into a 20-mL sample vial. A 65 μm PDMS/DVB fiber (Supelco, PA, USA) was exposed to the headspace of the vial at 50 °C for 25 min. After extraction, the fiber was directly desorbed into the injection port of the GC at 250 °C. The analytes were determined by GC/MS (GC, Agilent 7890B; MS, Agilent 5977A, Agilent, CA, USA) equipped with a methyl polysiloxane capillary column (HP-5MS, Agilent; 30 m × 0.25 mm × 0.25 μm film thickness). The carrier gas was helium at 1.0 mL/min. The GC column temperature procedure was set as follows: kept at 40 °C for 10 min, increased to 240 °C at 5 °C/min, increased to 280 °C at 20 °C/min, and held for 8 min. The MS operated in electron impact (EI) mode with EI energy of 70 eV; and collected data at a rate of 0.7 scans/s over a range of *m/z* 40–650. The VOCs were tentatively identified by the comparison of actual mass spectra with the published authentic spectra database in the GC/MS libraries (NIST2011), and the compounds with MS match index over 800 were reported.

### 3.8. FAAs Analysis

FAAs were determined according to Zhou et al. [39] with some modifications. Thereby, 5 g mashed turbot sample and 15 mL of 15% cold trichloroacetic acid were mixed and homogenized at 11,620 g for 5 min at 4 °C. After standing at 4 °C for 2 h, the homogenate was centrifuged at 5810× *g* for 15 min at 4 °C. Then, 5 mL supernatant was immediately neutralized to pH 2.0 and diluted with ultrapure water to 10 mL. The mixture was filtered through a 0.22 μm filter and analyzed by an amino acid analyzer (Hitachi L-8800, Tokyo, Japan). Peak identification and quantification were accomplished by determining the retention times and peak areas from the instrument software in comparison to FAA standards (Sigma, St. Louis, MO, USA).

### 3.9. Total Viable Count (TVC) Analysis

Representative 10 g minced muscle was aseptically homogenized with 90 mL of sterilized saline solution (0.85% NaCl) and then subjected to serial dilutions. TVC on nutrient agar medium (Hopebio, Qingdao, China) with 0.4 mg/mL nystatin (Aladdin, Shanghai, China) were incubated at 28 °C for 48 h.

### 3.10. Organoleptic Evaluation

The organoleptic properties of turbot samples were evaluated according to Li et al. [51]. Ten experienced judges (5 men and 5 women between 25 and 40 years old) participated in the organoleptic evaluation during each session. The panel was trained using turbot of different quality levels in each session. The panel trains involved training in the detection and recognition of odor, color, mucus, elasticity, and tissue morphology using a five-point scale. Thereby, 5 corresponded to ‘most liked’ and 1 to ‘most disliked’. In preparation for the organoleptic evaluation, two equal turbot portions (approximately 10 g per piece) were cut from the centre part of each turbot and placed in aluminium boxes coded with random three-digit numbers. Organoleptic analysis was conducted independently in a well-ventilated room with 20 ± 1 °C and 55 ± 2% relative humidity. The turbot samples were defined to have reached its maximum shelf-life when a rancid flavor or odor value <3 was obtained. At that value, most panelists detected the attribute at an intensity level that would deem the turbot unacceptable.

### 3.11. Statistical Analysis

Experimental data were analyzed using SPSS 22.0 (IBM Corporation, New York, NY, USA). The one-way ANOVA procedure followed by Duncan’s multiple range tests was adopted to determine the significant difference (*p* < 0.05) between treatment means, and the results were expressed as means ± SD of three independent experiments.

## 4. Conclusions

The MAP in combination with superchilling (−1.3 °C) treated turbot samples showed a potential in slowing down the rate of turbot spoilage. The MAP1 and MAP2 treatments maintained better physicochemical results, flavor quality, and organoleptic evaluation results during superchilling storage, which is mainly due to high CO_2_ MAP effectively inhibiting the growth of spoilage microorganisms. VP treatments combined with superchilling was also proven to be effective in controlling the microbiological growth. However, exudation and acid production render such packaging undesirable. MAP1 and MAP2 treatments had similar effects in slowing down turbot samples spoilage. According to organoleptic evaluation, MAP2 treatments led to a longer shelf life than MAP1 treatments for turbot samples during superchilling storage. Therefore, MAP2 (60% CO_2_/10% O_2_/30% N_2_) combined with superchilling (−1.3 °C) is suitable for maintaining the freshness of turbot samples where an extended storage period may be necessary.

## Figures and Tables

**Table 1:** Changes in FAA content (mg/100g) of turbot with different treatments during storage period.

Storage Time	Samples	FAAs								
		Asp	Thr	Ser	Glu	Gly	Ala	Val	Met	
Day 0		4.98 ± 0.12	8.92 ± 0.67	17.08 ± 1.51	9.23 ± 1.16	30.22 ± 0.14	30.2 ± 1.86	27.78 ± 4.24	2.68 ± 0.58	
Day 15	AP	13.33 ± 1.14 ab	10.82 ± 0.26 a	16.86 ± 1.04 a	16.76 ± 0.22 cd	35.27 ± 0.96 a	34.92 ± 0.66 a	28.01 ± 0.27 a	4.68 ± 0.23 cd	
	VP	10.09 ± 0.39 cd	10.26 ± 1.59 a	11.02 ± 0.45 c	20.59 ± 0.41 a	24.42 ± 0.55 d	26.85 ± 0.36 c	17.81 ± 0.24 b	7.11 ± 0.74 a	
	MAP1	13.25 ± 1.04 ab	8.71 ± 0.18 ab	11.13 ± 0.75 c	16.78 ± 0.29 cd	24.91 ± 0.02 d	26.16 ± 0.35 cd	12.06 ± 0.49 d	4.95 ± 0.1 c	
	MAP2	11.23 ± 0.85 c	10.73 ± 0.23 a	13.78 ± 1.29 b	20.88 ± 0.7 a	26.24 ± 0.04 b	23.13 ± 0.3 f	10.58 ± 0.61 e	3.91 ± 0.19 e	
	MAP3	16.24 ± 2.06 a	9.03 ± 2.18 ab	8.10 ± 0.36 e	19.36 ± 0.29 b	22.82 ± 0.42 e	25.21 ± 0.63 cde	8.91 ± 0.22 f	6.08 ± 0.09 b	
	MAP4	14.18 ± 2.03 ab	10.65 ± 2.19 a	9.4 ± 0.16 d	17.11 ± 0.32 e	25.01 ± 0.01 c	27.74 ± 0.29 b	14.36 ± 0.11 c	6.54 ± 0.15 a	
Day 27	MAP1	3.17 ± 0.36 b	9.58 ± 0.05 b	13.08 ± 0.12 b	12.46 ± 0.12 bc	16.46 ± 0.03 b	15.38 ± 0.28 d	23.51 ± 2.11 a	2.99 ± 0.53 a	
	MAP2	5.1 ± 0.13 a	12.26 ± 0.12 a	14.14 ± 0.07 a	18.86 ± 0.13 a	20.42 ± 0.18 a	18.84 ± 0.39 c	15.45 ± 1.03 b	2.34 ± 0.16 ab	
	MAP3	2.73 ± 0.18 bc	9.55 ± 0.08 b	9.56 ± 0.26 c	12.70 ± 1.15b c	10.20 ± 0.16 d	21.41 ± 1.25 ab	8.75 ± 0.11 d	2.67 ± 0.16 a	
	MAP4	1.79 ± 0.08 d	6.18 ± 0.15 c	5.34 ± 0.23 d	14.02 ± 0.35 b	13.23 ± 0.42 c	22.96 ± 0.32 a	11.18 ± 1.35 c	1.12 ± 0.13 c	
		**Ile**	**Leu**	**Tyr**	**Phe**	**Lys**	**His**	**Arg**	**Pro**	**Total**
Day 0		2.51 ± 0.41	4.52 ± 0.02	1.54 ± 0.68	4.09 ± 0.43	8±3.51	1.91 ± 0.55	2.99 ± 0.25	6.23 ± 0.15	162.88 ± 8.58
Day 15	AP	4.54 ± 0.5 e	5.79 ± 0.13 e	3.5 ± 0.6 d	6.88 ± 1.12 b	2.87 ± 0.16 e	9.65 ± 0.32 a	6.07 ± 0.43b c	9.8 ± 0.35 a	209.75 ± 5.39 a
	VP	7.35 ± 0.19 a	10.66 ± 0.05 a	6.83 ± 0.93 ab	10.06 ± 0.19 a	3.96 ± 0.37 c	8.23 ± 0.16 b	9.49 ± 0.86 a	8.14 ± 0.15 e	192.87 ± 3.99 b
	MAP1	5.27 ± 0.17 cd	6.5 ± 0.58 d	4.64 ± 0.24 c	4.73 ± 0.08 c	3.66 ± 0.24 cd	7.45 ± 0.11 c	6.21 ± 0.11b c	8.7 ± 0.16 c	165.11 ± 2.35 e
	MAP2	5.63 ± 0.33 c	3.54 ± 0.13 f	2.03 ± 0.28 e	3.98 ± 0.36 d	5.9 ± 0.07 b	4.98 ± 0.04 e	6.34 ± 0.14 b	9.23 ± 0.02 b	162.11 ± 3.13 ef
	MAP3	6.74 ± 0.7 ab	7.71 ± 0.31 bc	7.54 ± 0.25 a	7.14 ± 0.26 b	5.46 ± 0.51 b	6.14 ± 0.13 d	9.55 ± 0.26 a	8.42 ± 0.03 d	174.45 ± 5.2 d
	MAP4	7.2 ± 0.05 a	8.05 ± 0.13 b	6.54 ± 0.02 ab	6.83 ± 0.37 b	8.14 ± 0.13 a	6.24 ± 0.23 d	9.19 ± 0.03 a	8.64 ± 0.11 c	185.82 ± 3.47 bc
Day 27	MAP1	3.6 ± 0.26 a	5.4 ± 0.16 a	2.27 ± 0.13 bc	3.27 ± 0.41 a	1.6 ± 0.11 a	2.7 ± 0.2 a	4.18 ± 0.3 c	4.1 ± 0.26 cd	123.75 ± 5.43 b
	MAP2	2.67 ± 0.15 c	4.01 ± 0.12 c	3.87 ± 0.12 a	2.67 ± 0.38 ab	1.31 ± 0.09 b	2.02 ± 0.09 b	6.66 ± 0.27 ab	4.49 ± 0.32 c	135.11 ± 3.42 a
	MAP3	3.15 ± 0.23 ab	5.11 ± 0.52 ab	4.10 ± 0.38 a	1.93 ± 0.18 c	0.40 ± 0.09 d	1.92 ± 0.29 b	6.91 ± 0.36 a	7.11 ± 0.56 ab	108.2 ± 5.26 c
	MAP4	2.40 ± 0.13 cd	2.09 ± 0.28 d	2.70 ± 0.38 b	1.50 ± 0.32 cd	0.82 ± 0.15 c	1.20 ± 0.08 c	2.11 ± 0.16 d	8.40 ± 1.18 a	97.04 ± 3.38 d

Different lower case letters in different groups from the same day indicate significant differences (*p* < 0.05).

**Table 2:** Identification and semiquantification of main volatile compounds (peak area × 10^−6^) in turbot after 0, 15 and 27 days of the storage.

Compounds	0 d			15 d					27 d			Odor Description	References
		AP	VP	MAP1	MAP2	MAP3	MAP4	MAP1	MAP2	MAP3	MAP4		
**Alcohols**													
1-Penten-3-ol	4.06 ± 0.32A	7.59 ± 0.18 a,B	5.35 ± 0.3 b,B	5.84 ± 0.08 b,B	5.65 ± 0.17 b,B	6.03 ± 0.21 c,B	6.24 ± 0.13 d,B	10.63 ± 0.37 a, C	12.17 ± 0.14 b,C	15.26 ± 0.42 c,C	16.42 ± 0.28 d, C	Burnt, meaty, grassy-green	[30,31]
1-Octen-3-ol	4.7 ± 0.35A	7.06 ± 0.16 a,B	3.83 ± 0.46 b,B	6.39 ± 0.62 c,B	5.62 ± 0.27 d,B	5.69 ± 0.36 d,B	6.60 ± 0.57 e,B	13.63 ± 1.11 a, C	11.23 ± 0.3 b,C	17.45 ± 1.32 c,C	19.38 ± 1.63 d, C	Mushroom	[30,32]
2-Octyn-1-ol	8.67 ± 0.83A	ND	1.88 ± 0.16 a,B	1.05 ± 0.13 b,B	1.01 ± 0.06 b,B	1.21 ± 0.04 c,B	2.70 ± 0.32 d,B	ND	ND	1.82 ± 0.18 a,C	1.25 ± 0.15 b, C	Mushroom	[33]
(*Z*)-2-Penten-1-ol	ND	0.85 ± 0.13 a,A	0.73 ± 0.08 b,A	ND	0.93 ± 0.18 c,A	1.15 ± 0.36 d,A	1.28 ± 0.28 e,A	0.83 ± 0.15 a, B	1.32 ± 0.33 b,B	1.87 ± 0.19 c,B	1.68 ± 0.31 d, B	Green, plastic	[31,34]
1-Hexanol	2.63 ± 0.47A	0.46 ± 0.13 a,B	1.92 ± 0.27 b,B	2.38 ± 0.16 c,B	2.74 ± 0.38 d,B	1.85 ± 0.43 e,B	1.74 ± 0.2 ef,B	1.23 ± 0.18 a, C	1.52 ± 0.36 b,C	0.56 ± 0.16 c,C	0.47 ± 0.12 c,C	Grassy, woody, fatty	[31,35]
1-Heptanol	ND	1.49 ± 0.25 a,A	2.03 ± 0.3 b,A	1.86 ± 0.43 c,A	2.72 ± 0.56 d,A	1.56 ± 0.24 e,A	1.39 ± 0.37 f,A	ND	ND	ND	ND	Fresh, light green, nutty	[30,31]
2-ethyl-2-Hexen-1-ol	ND	ND	0.86 ± 0.23 a,A	0.61 ± 0.15 ab,A	ND	0.84 ± 0.17 b,A	1.36 ± 0.26 c,A	0.73 ± 0.19 a, B	ND	ND	0.61 ± 0.08 a,B	Citrus, floral, sweet	[35]
(*E*)-2-Octen-1-ol	ND	ND	0.85 ± 0.26 a,A	0.73 ± 0.17 b,A	0.68 ± 0.02 b,A	0.90 ± 0.32 c,A	0.63 ± 0.18 d,A	0.48 ± 0.12 a, B	0.53 ± 0.14 ab,B	0.36 ± 0.08 b,B	ND	Green	[36]
**Aldehydes**													
Hexanal	32.91 ± 3.23A	35.68 ± 2.8 a,B	28.2 ± 1.54 b,B	33.73 ± 3.62 c,B	30.54 ± 1.1 d,B	37.38 ± 2.38 e,B	36.87 ± 6.3 f,B	43.6 ± 3.85 a, C	39.69 ± 2.78 b,C	52.49 ± 4.83 c,C	55.38 ± 3.78 d,C	Fishy, grass	[30,32]
(*Z*)-4-Heptenal	0.68 ± 0.13 A	3.64 ± 0.83 a,B	2.18 ± 0.36 b,B	2.69 ± 0.28 c,B	2.35 ± 0.63 d,B	3.12 ± 0.35 e,B	3.08 ± 0.6 e,B	4.32 ± 0.78 a, C	5.59 ± 1.86 b,C	7.13 ± 2.03 c,C	6.83 ± 1.41 cd,C	Boiled potato, biscuit-like	[30,31]
2-methyl-butanal	4.63 ± 1.3 A	2.38 ± 0.58 a,B	3.12 ± 0.75 b,B	3.91 ± 1.25 c,B	3.62 ± 0.3 d,B	2.73 ± 0.47 e,B	2.54 ± 0.82 f,B	1.32 ± 0.38 a, C	0.87 ± 0.15 b,C	ND	ND	Green, almond, strong burnt,	[30,31]
Heptanal	6.59 ± 1.68 A	7.32 ± 1.36 a,B	5.87 ± 0.8 b,B	6.32 ± 0.75 c,B	6.79 ± 1.2 d,B	7.41 ± 1.56 e,B	7.63 ± 0.85 ef, B	8.78 ± 1.35 a, C	7.36 ± 0.99 b,C	9.33 ± 1.86 c,C	10.21 ± 2.38 d,C	Dry fish green, fatty, rancid	[30,32]
Benzaldehyde	5.72 ± 1.08 A	2.14 ± 0.49 a,B	5.36 ± 1.27 b,B	4.69 ± 1.56 c,B	5.87 ± 0.95 d,B	6.19 ± 1.61 e,B	5.08 ± 1.36 f,B	2.55 ± 0.68 a, C	2.73 ± 0.67 ab,C	2.38 ± 0.17 b,C	1.84 ± 0.37 c,C	Bitter almond, burnt sugar, woody	[30,32]
(*E*,*E*)-2,4-Heptadienal	5.54 ± 1.35 A	6.39 ± 2.3 a,B	ND	5.03 ± 0.47 b,B	4.72 ± 1.32 bc,B	5.78 ± 0.56 c,B	6.18 ± 1.78 d,B	5.69 ± 0.96 a, C	4.56 ± 1.32 b,C	7.03 ± 1.75 c,C	7.37 ± 1.68 d,C	Fatty, fishy, oxidized oil-like	[30,32]
Octanal	5.43 ± 1.3 A	10.73 ± 2.46 a,B	7.18 ± 1.83 b,B	7.47 ± 1.86 c,B	6.85 ± 0.85 d,B	8.03 ± 2.3 e,B	7.96 ± 1.76 f,B	13.60 ± 3.2 a, C	12.05 ± 1.86 b,C	16.72 ± 2.78 c,C	18.36 ± 3.36 d,C	Grassy, rancid, soapy, citrus	[30,32]
(*E*)-2-Octenal	1.22 ± 0.53 A	4.68 ± 1.12 a,B	2.38 ± 0.68 b,B	2.68 ± 0.79 bc,B	3.03 ± 1.02 c,B	4.19 ± 1.36 d,B	3.87 ± 0.88 e,B	6.95 ± 1.35 a, C	6.43 ± 2.13 b,C	8.18 ± 2.56 c,C	8.72 ± 2.78 d,C	Aromatic, oxidized oil-like	[30,32]
Nonanal	9.82 ± 2.36 A	2.03 ± 0.63 a,B	2.29 ± 1.02 b,B	1.63 ± 0.23 c,B	2.03 ± 0.22 d,B	2.81 ± 1.08 e,B	1.61 ± 0.02 f,B	1.56 ± 0.06 a,C	1.05 ± 0.32 b,C	0.72 ± 0.04 c,C	0.92 ± 0.31 d,C	Gravy, green, floral, waxy, soapy, fatty, citrus fruit	[30,32]
(*E*,*Z*)-2,6-Nonadienal	0.52 ± 0.13 A	1.78 ± 0.58 a,B	0.63 ± 0.23 b,B	0.42 ± 0.08 c,B	0.86 ± 0.19 d,B	1.36 ± 0.36 e,B	1.52 ± 0.72 f,B	ND	ND	1.53 ± 0.2 a,C	1.68 ± 0.62 ab,C	Cucumber-like, fatty, green	[31,37]
Decanal	1.47 ± 0.35 A	0.38 ± 0.17 a,B	0.43 ± 0.18 b,B	0.54 ± 0.13 c,B	0.43 ± 0.2 cd,B	0.53 ± 0.18 d,B	0.38 ± 0.12 de,B	ND	ND	ND	ND	Citrussy	[37]
2,3-Octanedione	2.58 ± 1.36 A	8.62 ± 2.11 a,B	3.86 ± 1.23 b,B	4.78 ± 1.26 c,B	5.03 ± 1.38 d,B	6.83 ± 1.82 e,B	6.61 ± 1.37 ef,B	8.63 ± 2.32 a,C	10.39 ± 1.78 a,C	14.27 ± 2.85 a,C	13.69 ± 3.3 a,C	Oxidized fat	[38]
(*E*,*E*)-3,5-Octadien-2-one	4.42 ± 1.37 A	1.75 ± 0.29 a,B	1.25 ± 0.26 b,B	ND	0.97 ± 0.23 c,B	1.36 ± 0.47 d,B	0.83 ± 0.35 bc,B	ND	0.63 ± 0.17 a,C	1.08 ± 0.28 b,C	ND	Fruity, grassy, mushroom	[31,39]
2-Undecanone	1.39 ± 0.48 A	1.89 ± 0.79 a,B	1.37 ± 0.28 b,B	1.63 ± 0.74 c,B	1.47 ± 0.05 cd,B	1.58 ± 0.38 cd,B	1.46 ± 0.52 cd,B	0.78 ± 0.3 a,C	0.53 ± 0.18 b,C	0.73 ± 0.23 ab,C	0.85 ± 0.35 c,C	Fruity-rosy, orange-like	[31,39]
2,3-Pentanedione	ND	1.65 ± 0.26 a,A	0.45 ± 0.17 b,A	0.84 ± 0.23 c,A	1.05 ± 0.18 d,A	1.48 ± 0.36 a,A	1.26 ± 0.25 dA	ND	ND	1.84 ± 0.73 a,B	0.76 ± 0.11 b,B	Butter scotch, almond, fruity	[31]
3-Pentanone	ND	2.54 ± 0.6 a,A	ND	1.68 ± 0.15 b,A	2.32 ± 0.38 a,A	3.15 ± 1.37 c,A	2.78 ± 0.86 d,A	2.63 ± 1.02 a,B	2.08 ± 0.77 b,B	5.74 ± 2.3 c,B	ND	Irritant, acetone	[32]

ND: Not detected. Different uppercase letters in the same group from different day indicate a significant difference (*p* < 0.05). Different lowercase letters in different groups from the same day indicate a significant difference (*p* < 0.05).
